# Iowa gambling task: Administration effects in older adults

**DOI:** 10.1590/S1980-57642008DN10100011

**Published:** 2007

**Authors:** Daniela Di Giorgio Schneider, Gabriela Peretti Wagner, Natalie Denburg, Maria Alice de Mattos Pimenta Parente

**Affiliations:** 1 Universidade Federal do Rio Grande do Sul (UFRGS), Instituto de Psicologia, Programa de Pós-Graduação em Psicologia –Psychology MD (UFRGS).; 2 Division of Cognitive Neuroscience, Department of Neurology, University of Iowa Carver College of Medicine, #2007 RCP, 200 Hawkins Drive, Iowa City, Iowa52242-1053 USA.; 3 Universidade Federal do Rio Grande do Sul (UFRGS), Instituto de Psicologia, Programa de Pós-Graduação em Psicologia – Psychology PhD (USP).

**Keywords:** tomada de decisão, Iowa Gambling Task, envelhecimento

## Abstract

The Iowa Gambling Task (IGT) assesses decision-making.

**Objective:**

The objective of the present study was to investigate whether specific changes in administering the IGT can affect performance of older adults completing the task.

**Method:**

Three versions of the IGT were compared regarding the feedback on the amount of money won or lost over the course of the test. The first version (I) consisted of a replication of the original version (Bechara et al., 1994), which utilizes a computerized visual aid (green bar) that increases or decreases according to the gains or the losses. The second version (II), however, involved a non-computerized visual aid (cards) and, in the third version (III) the task did not include any visual aid at all. Ninety-seven older adults, divided into three groups, participated in this study. Group I received computerized cues (n=40), group II, non-computerized cues (n=17) and III was submitted to a version without any cues (n=40).

**Results:**

The participants without any cues achieved only a borderline performance, whereas for those with non-computerized cues, twice the number of participants showed attraction to risk in relation to those with aversion. The participants of the computerized version were homogeneously spread across the three performance levels (impaired, borderline and unimpaired).

**Conclusions:**

Aspects of the complexity of the decision process as well as of the task used are proposed as possible theoretical explanations for the performance variation exhibited.

## Abstract (pt)

A Iowa Gambling Task (IGT) consiste em um instrumento que avalia tomada de decisão.

**Objetivo:**

O objetivo do presente estudo foi investigar se alterações específicas na administração da IGT podem afetar o desempenho de idosos na execução da tarefa.

**Método:**

Três versões da IGT foram comparadas, de acordo com o resultado obtido em termos do número de cartas selecionadas de cada um dos baralhos. A primeira versão (I) consistiu em uma replicação da versão original (Bechara et al., 1994), a qual apresenta uma pista visual (barra verde) que aumenta ou diminui de acordo com ganhos e perdas monetários. A segunda versão (II) envolveu uma pista visual não computadorizada (cartões) e, na terceira versão (III), não houve nenhuma espécie de pista. Noventa e sete idosos, divididos entre os três grupos, participaram do estudo. O grupo I (pista computadorizada) foi composto por 40 idosos, enquanto os grupo II (pista não computadorizada) contou com 17 e, por fim, o grupo III (sem pista), foi formado por 40 idosos.

**Resultados:**

Os participantes submetidos à versão sem pista apresentaram um desempenho limítrofe. Em relação aos idosos submetidos à variação com pista não computadorizada, os participantes apresentaram duas vezes mais atração ao risco, quando comparados àqueles com aversão. Os participantes que realizaram a tarefa com pista computadorizada apresentaram desempenho homogêneo entre os três subgrupos de desempenho quanto à busca ou aversão pelo risco.

**Conclusões:**

Possíveis explicações teóricas das diferenças de desempenho decorrem principalmente de diferentes aspectos da complexidade do processo de decisão e da tarefa.

Over the last decade, the *Iowa Gambling Task* (IGT)^1^ has been widely used among researchers as a neuropsychological measure that assesses decision-making (DM) under ambiguity. This task intends to simulate a real-life decision-making situation, in which the participants have to choose cards in order to win or lose money. The task enables classification of a subject’s decision-making behavior in terms of risk aversion versus risk taking.

The IGT was first developed to investigate the realworld deficits exhibited by patients with lesions in the ventromedial prefrontal cortex (VMPC)^1-4^. Prior to the development of the IGT, conventional “lab” tasks were not able to index the types of defective decisions such patients took in their everyday-life. Briefly, patients with lesions to the VMPC displayed myopia for the future, preferring short-term reinforcement over long-term repercussions. Despite strong evidence for the IGT’s sensitivity to VMPC functioning, some studies have indicated that the IGT may present some limitations as regards its lack of specificity. In other words, studies carried out on patients that have damage in non-VMPC brain areas have also showed impairment in their ability to decide^5^.

One of the hypothetical explanations places emphasis on the IGT’s complexity, an aspect that requires the involvement of other cognitive components besides decision- making. A first process that seems to be of special relevance concerns behavioral inhibition, crucial for a decision process to take place^6^. In other words, in order to allow a balance between different options of choice, it is necessary to control impulsive behavior enabling appropriate use of the cognitive process for the information handled. Thus, attention processes seem to be equally important, enabling relevant aspects of the information on the options to be highlighted^7^. Similarly, the information regarding these options must be held in the conscious process for some time using the working memory, allowing the use of reasoning processes and judgment concerning the advantages and disadvantages of each option of choice.

Therefore, it is plausible to assume that particular administration differences amongst the different IGT versions used, such as the kind of financial feedback provided or reinforcement used (real or fake money), among others, may be an important factor to consider. The underlying reason behind this is that these differences may provide additional important hints that can maximize the participation of some cognitive components much more than others, or vice versa. For instance, visual feedback can be memorized differently to verbal feedback, interfering in the memorization process necessary for appropriate performance of the task.

Some studies have focused on aspects of administering the task, which vary according to researcher, aiming to verify whether they can differentially affect the performance of the participants on the IGT. No difference has been observed regarding the kind of reinforcement used (real or facsimile money)^8-9^. Similarly, no difference in performance has been found stemming from the kind of version used (computerized or manual)^10^.When focusing these discussions on the elderly population^11-15^, the hypothesis over the possible effects of administration differences takes into consideration specific aspects of the ageing process. Regarding the cognitive changes found in this elderly population, we have considered segmented studies on memory loss, mainly those showing significant working memory damage and impaired ability to remember^11^. Thus, it is plausible to suggest that usage changes that facilitate the mnemonic process can assist older individuals in their decision-making process throughout the IGT.

Denburg, Tranel & Bechara^15^ carried out a study in which they observed that a sub group of older adults showed DM impairment compared to younger individuals. Moreover, when repeating the performance analysis of the elderly, the study by McPherson, Phillips & Della Sala^12^, Denburg et al.^15^ suggest that the same subgroup composition could be found if the authors had conducted a group subdivision. On the other hand, the study carried out by Schneider^16^ reveals that neither young nor old people showed evidence of borderline performance when submitted to a different version of the IGT. Basically, this version shares the same characteristics found in the original^1^, except for a lack of visual cues concerning the amount of gain and loss throughout the games (green bar, which increases and decreases). Wagner^17^ showed that the aid of a manual visual cue, which interfered in the decision processes of the older adults, improved the performance in terms of aversion to risk.

Considering the arguments presented so far, we note the importance of carrying out studies with different versions of the IGT to verify in what way the specific changes in task administration may affect individuals’ performance. Also, studies of this nature enable a better understanding about the different components involved in the task, allowing greater control of its complexity and a consequent increase in its specification. In this sense, the present study will compare three versions of the IGT regarding the feedback provided by the test in relation to the amount of money lost or won during the game. The first version (I) consists of a replication of the original version^1^. It has a computerized visual aid which is presented by means of a green bar which increases or de-creases according to the gains or losses. The second version (II) however, will involve a non-computerized visual aid (cards), which will enable the participants to have some idea of the amount gained (or lost) at any given time. In the third version (III), the task will not present any visual aid, thus not offering any idea of the amount won or lost at any given time.

## Methods

### Design and participants

Older adults were recruited from the community, and randomly assigned to one of the three aforementioned groups. Social-demographic data of the participants are provided in Table 1.Three groups of participants were evaluated. Group I had 40 participants (22 male), while there were 17 participants in Group II (all female) and 40 in Group III (13 male). The first group comprised Americans while the other two groups were Brazilians. According to the Kruskal-Wallis Test, there were no significant differences (p value greater than 0.05) among the groups concerning age [χ^2^(0.610)=2; p=0.737] or education [χ^2^(5.030)=2; p=0.081].

**Table 1:** Mean and standard deviation of age and education of the sample studied.

	Group I (computerized cue)	Group II (non-computerized cue)	Group III (without cue)
	n=40	n=17	n=40
Age	69.68 (8.27)	70.59 (8.16)	68.60 (5.02)
Education	15.52 (2.85)	14.94 (5.54)	13.80 (2.98)

Exclusion criteria involved non-corrected visual and/or hearing impairment, presence of psychiatric disorders (that required treatment or affect daily life), and/or neurological disease (vascular-brain disease, epilepsy, among others, including disorders that can generate changes in cognitive functions). Regarding the inclusion criteria, the minimum age was 56 years old, and participants must have received at least 4 years of formal education.

### Measures and procedures

The older adults were invited to participate in the study as volunteers. All the participants lived in the community, and had never been institutionalized. After being informed about the objectives of the study and having signed the Consent Agreement, the participants were submitted to evaluation.

### Self-report questionnaire of social-demographic and cultural data

Firstly, the Brazilian older adults completed a socialdemographic and cultural data self-report questionnaire, in order to obtain data to characterize the sample studied. Through this form it was possible to glean information such as age, education, social-economic level, reading and writing habits, among others. The data on demographic variables were obtained through objective questions. Regarding the North American sample, the participants were submitted to a semi-structured interview^15^, which had similar objectives to those in the Brazilian sample form.

### Iowa gambling test (IGT)

The IGT is a neuropsychological measure that assesses decision-making ability (1). The computerized version was used in all three studies, with some variations, as outlined below. All of the statistical data were analyzed with SPSS, version 12.

### Original version (with computerized visual reinforcement)^1^

Using monetary choices, the task allows classification of the participant’s performance in terms of aversion or risk taking. The task consists of four decks of cards of similar appearance (labeled A, B, C and D). The participant is told, during the initial verbal instructions, to select one card at a time from any of the fours decks. They are told that each time they choose a card they will win some money and that occasionally they will also lose some money. They are also told that the goal of the IGT is to win money, or maximize profit.

For instance, when the participant chooses a card from Decks A and B, they win R$ 100,00, whereas when they choose from Decks C and D, they might win R$ 50,00. Decks A and C have frequent but low penalties whereas Decks B and D have occasional penalties but of high values. Despite the large amount of money one can win, Decks A and B are considered disadvantageous because the losses outweigh the gains, yielding an overall net loss. However, Decks C and D are considered advantageous, because the gains outweigh the losses, yielding an overall net gain. The task continues until all 100 cards are selected. During the task, the individual can obtain an estimate of the value won so far, but cannot know the amount exactly. This is possible because the computer offers a cue informing the participant about the losses and gains. Specifically, there are two bars are displayed on the computer screen, one that does not change representing the initial situation (the loan the participant receives), and another indicating the gains and losses, that increases or decreases in size as the wins and losses are accumulated. As the participant performs the task, this bar provides information (not exact numbers) of the remaining monetary balance.

### Version with non-computerized visual reinforcement^17^

This task is the same as the original version^1^, with one variation. At every selection, besides the message of receiving or losing money on the computer screen, the participant gains or loses the equivalent in colorful tokens presented by the examiner. The tokens used are of three colors: yellow (worth R$ 25,00), blue (worth R$ 50,00), and pink (R$ 100,00 each).

The older adults were not informed about how much each token was worth, only that they represent money won by them during the test. As money is lost or won, tokens of the corresponding values are placed into or withdrawn from sight of the participant. The participants were not told to make precise calculations of losses or gains. The objective was that only an estimate of the balance was available, from the accumulation or loss of colorful tokens. The indexes used for the calculations were the same as in the original version.

### Version without visual reinforcement^16-18^

This version without visual reinforcement was adapted by Schneider^16^, and is administrated according to standard protocol^10^, except for one aspect. In this version, the participants had no computerized bars with the direction of the gains and the losses, where this aid is believed to facilitate the development of an approximation of the value obtained throughout the experiment. The participants received computerized feedback related to the amount lost or gained, without, however, receiving the mnemonics given by the green bar, which increased or decreased according to the losses and gains. Thus, participants were informed of the wins and potential losses for each card choice, but were not provided a directional visual depiction of cumulative gains and losses.

### Calculation of the aversion to risk and classification of the participants

The same calculation of the aversion to risk was used in the three versions studied, and this served as the main dependent variable. From the result of choices from the four decks we were able to establish an index through the following operation [(C+D)-(A+B)]. The performance of each participant was classified after the calculation according to the criteria of Denburg et al.^15^, under of the three following categories:

(1) *Participant unimpaired* (aversion to risk);
(2) *participant borderline*; and
(3) *participant impaired (risk attraction)*.

Scores below zero indicate loss of money and risk attraction, whereas scores greater than zero indicate gain of money and an aversion to risk. The Chi-square test (χ^2^) was used to calculate data related to the *Iowa Gambling Test* (aversion to risk).

### Ethical aspects

Participants in each of the three groups completed an evaluation according to the bioethical norms governing research on humans. For Brazilian participants, these rights were assured according to norms^19-20^. The research project related to group II went before the Research Ethics Committee of the Universidade Federal do Rio Grande do Sul (CEP/UFRGS) and was registered under number 2005463. This was approved at meeting number 43, of 24/11/2005, minute nº 64, and deemed ethically, and methodologically adequate^20^.

Regarding the North American participants, they were invited to participate in the research, and given the choice whether they would like to take part in the study or not. If they agreed, they would receive the Informed Consent Document, which included the purpose of the study in detail. Additionally, the participants were paid for taking part in this research study ($12.50 per hour). All the research assistants completed the online course “Human Participant Protections Education for Research Teams”, sponsored by the National Institute of Health (NIH).

## Results

The data related to the *Iowa Gambling Test* (calculations of aversion to the risky decks) were analyzed using the Chi-square (χ^2^) test. As can be seen from the data provided in Table 2, the participants without any cues had only borderline performance, whereas out of those with non-computerized cues, twice the number of participants showed attraction to risk than those with aversion. The participants of the computerized cue version were homogeneously spread across the three groups (Impaired, borderline and unimpaired performance). The analysis was firstly performed using the test of Chisquare. The distribution of the three groups was significantly different (χ^2^=40, 99 df=4, p value less than 0.001). The expected value corresponds to 10, which enables comparison through the Chi-square, in spite of the two occurrences lower than 5^21^. Since this position does not extend to the statistic studies^22^, a second comparison grouped the borderline patients with the patients with attraction to risk. The Fisher’s exact test showed a significant difference between the cueless version compared to both the computerized cue version (p<0.001) and the non-computerized cue version (p<0.001). Thus, even with a smaller group receiving no computerized cues, significant differences were obtained. However, the computerized cue version did not differ significantly from the non-computerized one (p=0.61).

**Table 2:** Number of participants with or without aversion to risk in the three groups.

	No cue	Non-computerized cue	Computerized cue	Total
Impaired	0	6	14	20
Borderline	40	8	13	61
Unimpaired	0	3	13	16
Total	40	17	40	97

**Figure 1:**
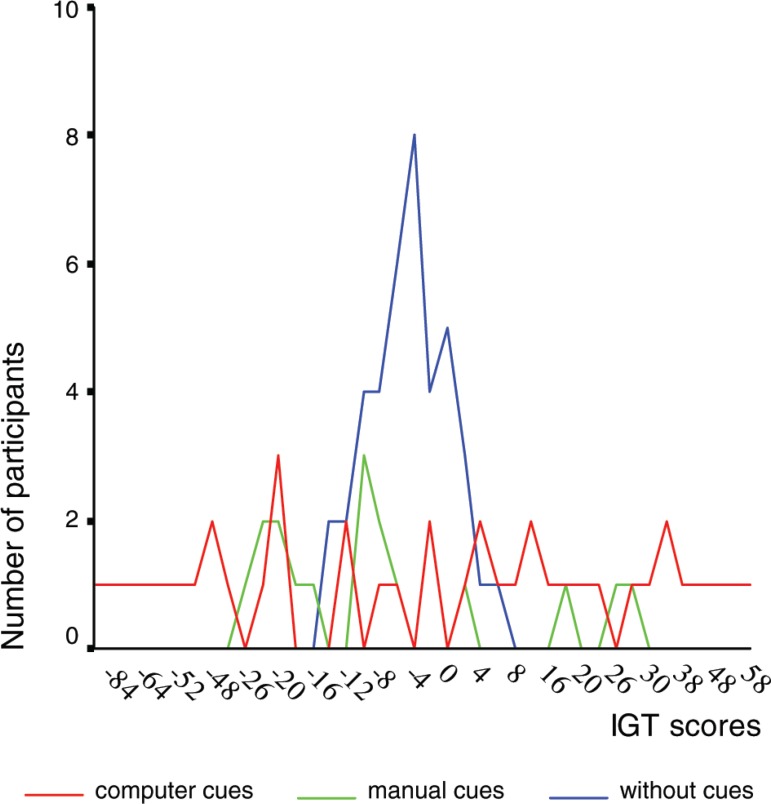
Graph of the dispersion of the three groups of elderly.

Figure 1 shows the individual scores of each participant submitted to one of the three versions of the experiment. We can observe that the computer version stimulated a wider range of scores from –84 to + 64, whereas the manual cue showed a more restricted range, with a slightly greater number of participants having negative scores. The group without cues, on the other hand, resulted in little variation in the scores of participants, showing a curve in a central position near zero. In other words, the computerized cue version lead to the choice of one of the tendencies, while the version without cues with the result around zero indicates that the participants made similar choices for advantageous decks as well as disadvantageous.

## Discussion

In the present study, the participants in the *computerized cue version*, version I, were spread more homogenously across the three subgroups of performance classification, that is, one third showed impaired performance. For group II, *non-computerized visual reinforcement*, in spite of the fact that the majority had their performance classified as borderline, twice as many participants showed impaired performance in relation to those with aversion to risk. The older adults belonging to group III, without cue version, showed borderline performance in relation to aversion to risk, as their behavior did not significantly differentiate from the value “0.”

The first explanation for the difference in performance among the groups is related to the complexity of the process in the decision-making and consequently of the IGT itself, that could have stemmed from administration differences. decision-making is a complex cognitive process, which involves other cognitive components throughout. Given the IGT involves these components to measure the decision process, any alteration in instructions might decrease the role of some specific and hierarchal subordinated cognitive processes, such as working memory, explicit learning^23^ and attention, minimizing the influence of these in the process of making choices during the task. Although not addressed in the present study, motivation might also play an important role in the performance on the IGT^24^.

Empirical evidence corroborates the notion of the involvement of other cognitive components in DM. Besides the emotional factors clearly presented in the somatic marker hypothesis^7^, DM involves the learning processes (through association between specific categories of stimuli and specific categories of somatic states), attention processes and working memory components, making this outcome feasible^6,7,25,26^.

The working memory acts by aiding the temporary holding of information concerning different options of choice in conscious processing, allowing processes of logical thinking and reasoning about advantages and disadvantages of each option to be employed. The working memory is also responsible for the challenging task of considering past events and projecting them to a scenario in the future, based on the present situation^6^.

On submission to the IGT, the cognitive and emotional information that has already passed is reactivated when the individual considers the characteristics involved in each deck, contributing to the analysis of the actual chances. Attention processes seem to be also required, enabling those relevant aspects of the information of choices to be highlighted^7^.

In this sense, it is plausible to consider that the introduction of a reinforcement cue albeit visual or computer based, authorizes the allocation of more cognitive resources for the decision. Thus, the use of a cue would facilitate the task, especially amongst the aged, by allowing lesser interference of the cognitive, attention, and mnemonic processes in the decisional process.

Although some studies investigating the role of the working memory in the performance of individuals on the IGT have presented contradictory results^27-30^, an alternative explanation is proposed. This proposes that the working memory and decision-making are asymmetrically dependent, evidencing a simple dissociation^3,25,31,32^. This implies the working memory is not dependent on impairment in decision-making, in other words, participants can present a working memory in the presence or absence of a deficit in decision-making. However, integrity of decision-making seems to depend on a working memory without impairment.

Revisiting the model of the somatic markers, it is valid to consider that beyond the attention and mnemonic processes involved in the judgment and analysis of the choice options, as well as in decision-making, the emotional learning that the individual develops during the course of the task makes it equally important. Thus, besides involving cognitive processes usually accessed by conventional tests that measure executive powers, the IGT calls on participants’ feelings regarding the options, which can be characterized as an emotional anticipation of the consequences^33^. Kahneman^34^ follows a similar line, when defending two cognitive function approaches: an intuitive way involving fast and automatic decision-making, implicitly, and a controlled side, which involves a slower and pondered process.

Regarding the performance of the individuals on the IGT, when the necessity of resources of working memory is minimized, the role that this would assume long ago, mainly in the aged population, seems to be reduced. In other words, if when offering a visual cue of the value gained (or lost) at the time one gives the elderly more information regarding the task, the work of the calculation during the course of the process is facilitated. Hence, it can be inferred that a more controlled learning is facilitated, since these cognitive processes are less vulnerable to error, increasing the role of the most implicit processes when choosing the decks.

This hypothesis requires further studies verifying its assertions empirically. Firstly, because even though the alterations in the instruction of the task may have reduced the role of some resources of memory and attention, much of these are still necessary for a satisfactory result. Furthermore, the notion that a greater role of the implicit components would have led the aged to demonstrate a higher attraction to risk contradicts the ideas of some empirical evidences demonstrating a preserved emotional process of positive and negative valences in this population, compared with the young^13,35^.

Amongst other possible variables involved in the choice of the process, it is reasonable to believe that characteristics of personality exert a certain tendency in preferences. Besides the related cognitive and emotional processes, some studies have shown that characteristics of personality can have some effect on the choices made, if added to the influences of the somatic marker and the executive processes^36-38^.

In addition to the complexity of the process of DM, a second, nevertheless no less important explanation for the results involves the possible influence of cultural aspects on the performance of the individuals in the different versions. Some authors have shown the influence of factors such as education and partner-cultural level in different neuropsychological tests, as well as those that evaluate executive powers^39^, attention^40^, perception^41^, memory^42^, language^43^ and constructive praxis^44^. However, in the case of the IGT, the importance of gambling habits and values of a particular culture may influence the greater or lesser tendency for risk. Therefore, the continuity of relevant studies comparing the same version of the task in different cultures is important.

Finally, it has been verified that there might be many variables involved in a decisional process, as measured by the IGT. Additionally, consensus is lacking among researchers on how much each variable is essential for the learning developed in the task.More studies are necessary controlling the different variables involved in the performance of individuals on the IGT, in order to better understand the process, thereby improving its specificity as a neuropsychological measure of DM.Moreover, further studies must be conducted, focusing on the validation process of the different versions, especially the version with non-computerized reinforcement. Given the demographic differences observed in the Brazilian population, the investigation of such effects on individuals’ performance on the IGT may be an important direction for the continuity of this research.

## References

[1] Bechara A, Damasio AR, Damasio H, Anderson S (1994). Insensitivity to future consequences following damage to human
prefrontal cortex. Cognition.

[2] Bechara A, Damasio H (2002). Decision-making and addiction (part I): impaired activation of
somatic states in substance dependent individuals when pondering decisions
with negative future consequences. Neuropsychologia.

[3] Bechara A, Damasio H, Damasio AR (2000). Emotion, decisionmaking, and the orbitofrontal
cortex. Cereb Cortex.

[4] Tranel D, Bechara A, Denburg N (2002). Asymmetric functional roles of right and left ventromedial
prefrontal cortices in social conduct, decision-making and emotional
processing. Cortex.

[5] Fellows LK, Farah M (2003). Different underlying impairments in decision-making following
ventromedial and dorsolateral frontal lobe damage in humans. Cereb Cortex.

[6] Palmini A, Knapp P (2005). O cérebro e a tomada de decisões. Terapia cognitivo-comportamental na prática
psiquiátrica.

[7] Damasio A (1996). O Erro de Descartes.

[8] Bowman CH, Turnbull OH (2003). Real versus facsimile reinforces on the Iowa Gambling
Task. Brain Cogn.

[9] Mazas CA, Finn PR, Steinmetz JE (2000). Decision-making biases, antisocial personality, and early-onset
alcoholism. Alcohol Clin Exp Res.

[10] Bechara A, Tranel D, Damasio H (2000). Characterization of the decision-making deficit of patients with
ventromedial prefrontal cortex lesions. Brain.

[11] Parkin AJ, Parkin A.J (1993). Getting old. Memory phenomena, experiment and theory.

[12] MacPherson SE, Phillips LH, Della Sala S (2002). Age, executive function, and social decision-making a
dorsolateral prefrontal theory of cognitive aging. Psychol Aging.

[13] Wood S, Busemeyer J, Koling A, Cox C, Davis H (2005). Older adults as adaptive decision-makers evidence from the Iowa
Gambling Task. Psychol Aging.

[14] Kovalchik S, Camerer C, Grether D, Plott C, Allman JM (2005). Aging and decision-making a comparison between neurologically
healthy elderly and young individuals. J Econ Behav Org.

[15] Denburg N, Tranel D, Bechara A (2005). The ability to decide advantageously declines prematurely in some
normal older persons. Neuropsychologia.

[16] Schneider DDG (2004). O desempenho de adultos jovens e idosos na tarefa do jogo: um estudo
sobre tomada de decisão.

[17] Wagner GP (2006). Disfunções executivas no envelhecimento cognitivo:
investigações com os instrumentos tarefa do jogo e teste
Wisconsin de classificação de cartas.

[18] Schneider DDG, Parente MAMP O desempenho de adultos jovens e idosos no Iowa Gambling Task: um
estudo sobre a tomada de decisão. Psicologia: Reflexão e Crítica.

[19] CFP Conselho Federal de Psicologia (2000). Resolução 016/2000. Resolução para pesquisa
com seres humanos.

[20] Conselho Nacional de Saúde (1996). Resolução 196/1996. Diretrizes e normas regulamentadoras
de pesquisas envolvendo seres humanos.

[21] Callegari-Jacques SM (2004). Bioestatística. Princípios e
aplicações.

[22] Dancey CP, Reidy J (2006). Estatística sem matemática para psicologia.

[23] Gutbrod K, Krouzel C, Hofer H, Muri R, Perrig W, Ptag R (2006). Decision-making in amnesia: Do advantageous decisions require
conscious knowledge of previous behavioural choices?. Neuropsychologia.

[24] Czernecki V, Pillon B, Houeto JL, Pochon JB, Levy R, Dubois B (2002). Motivation, reward, and Parkinson's disease: Influences of
dopatherapy. Neuropsychologia.

[25] Bechara A, Damasio H, Tranel D, Anderson C (1998). Dissociation of working memory from decision-making within the
human prefrontal cortex. J Neurosci.

[26] Damasio A (2003). Looking for Spinoza: joy, sorrow and the feeling brain.

[27] Crone EA, van der Molen MW (2004). Developmental changes in real life decision-making: performance
on a gambling task previously shown to depend on the ventromedial prefrontal
cortex. Dev Neuropsychol.

[28] Hinson JM, Jameson TL, Whitney P (2002). Somatic markers, working memory, and
decision-making. Cogn Affect Behav Neurosci.

[29] Hinson JM, Jameson TL, Whitney P (2003). Impulsive decisionmaking and working memory. J Exp Psychology: Learn Mem Cogn.

[30] Hooper CJ, Luciana M, Conklin HM, Yarger RS (2004). Adolescents' performance on the Iowa Gambling Task: implications
for the development of decision-making and ventromedial prefrontal
cortex. Dev Psychol.

[31] Bechara A, Martin EM (2004). Impaired decision-making related to working memory deficits in
individuals with substance addictions. Neuropsychology.

[32] Martin EM, Pitrak DL, Weddington W (2004). Cognitive impulsivity and HIV serostatus in substance dependent
males. J Int Neuropsychol Soc.

[33] Turnbull OH, Evans CEY, Bunce A, Carzolio B, O'Connor J (2005). Emotion-based learning and central executive resources: an
investigation of intuition and the Iowa Gambling Task. Brain Cogn.

[34] Kahneman D (2003). A perspective on judgment and choice. Am Psychol.

[35] Denburg N, Buchanan T, Tramel D, Adolphs R (2003). Evidence for preserved emotional memory in normal older
persons. Emotion.

[36] Davis C, Patte K, Tweed S, Curtis C (2007). Personality traits associated with decision-making
deficits. Pers individ. differ.

[37] Franken IHA, Muris P (2005). Individual differences in decisionmaking. Pers individ differ.

[38] Gonzalez R, Vassileva J, Bechara A, Grbesic S, Sworowski L, Novak RM (2005). The influence of executive functions, sensation seeking, and HIV
serostatus on the risky sexual practices of substance dependent
individuals. J Int Neuropsychol Soc.

[39] Plumet J, GIL R, Gaonac'h D (2005). Neuropsychological assessment of executive functions in women:
Effects of age and education. Neuropsychology.

[40] Rosselli M, Tappen R, Williams C, Salvatierra J (2006). The relation of education and gender on the attention items of
the Mini- Mental State Examination in Spanish speaking Hispanic
elders. Arch Clin Neuropsychol.

[41] Herrera-Guzman I, Pena-Casanova J, Lara JP, Gudayol- Ferre E, Bohm P (2004). Influence of age, sex, and education on the visual object and
space perception Battery (VOSP) In a Healthy Normal Elderly
Population. Clin Neuropsychol.

[42] Johnson DK, Storandt M, Balota DA (2003). Discourse analysis of logical memory recall in normal aging and
in dementia of the Alzheimer type. Neuropsychology.

[43] Lecours A, Mehler J, Parente MA (1987). Illiteracy and brain damage - 1. Aphasia testing in culturally
contrasted populations (control subjects). Neuropsychologia.

[44] Ostrosky-Solís F, Ardila A, Rosselli M (1999). NEUROPSI: a brief neuropsychological test battery in Spanish with
norms by age and educational level. J Int Neuropsychol Soc.

